# Supplementary material for: Structure and mechanism of the Mrp complex, an ancient cation/proton antiporter
Source: eLife. 2020 Jul 31;9:e59407. doi: 10.7554/eLife.59407 (PMC7419157; doi:10.7554/eLife.59407)
Supplement: Supplementary file 1. [file elife-59407-supp1.docx]

**Supplementary Table 1**

**Residues built in the Mrp model.**

All side-chains are assigned, there are no UNK residues.

| Subunit | Number of residues in sequence | Residues built | Missing fragments |
| --- | --- | --- | --- |
| MrpA | 821 | 29-816 | N- and C-terminus |
| MrpB | 140 | 3-140 | N-terminus |
| MrpC | 110 | 1-107 | C-terminus |
| MrpD | 490 | 1-489 | C-terminus |
| MrpE | 158 | 1-158 | none |
| MrpF | 91 | 2-89 | N- and C-terminus |
| MrpG | 119 | 8-111 | N- and C-terminus |

**Supplementary Table 2**

**Homologous counterparts among Mrp (*Anoxybacillus flavithermus* WK1), complex I (*Thermus thermophilus*) and MBH (*Pyrococcus furiosus*)**

| **Proposed Mrp Module** | **Mrp complex** | **MBH** | **Module of MBH** | **Complex I** | **Module of CI** |
| --- | --- | --- | --- | --- | --- |
|  | - | - |  | Nqo1 | N-module |
|  | - | - |  | Nqo2 | N-module |
|  | - | - |  | Nqo3 | N-module |
|  | - | MbhL | MA hydrogenase | Nqo4 | Q-module |
|  | - | MbhK | MA hydrogenase | Nqo5 | Q-module |
|  | - | MbhN | MA hydrogenase | Nqo9 | Q-module |
|  | - | MbhJ | MA hydrogenase | Nqo6 | Q-module |
|  | - | MbhM | MA hydrogenase | Nqo8 | P-module |
|  | - | MbhI N-  terminal |  | Nqo7 N-terminal | P-module |
|  | - | MbhI C-  terminal |  | Nqo12 C-terminal |  |
| Proton translocation | MrpA TM1-TM16 | - |  | Nqo12 TM1-TM16 | P-module |
| Sodium translocation | MrpA TM17-TM19 | MbhD | Proton translocation? | Nqo10 | P-module |
| Sodium translocation | MrpA TM20 – TM21 | MbhE | Proton translocation? | Nqo10 | P-module |
| Sodium translocation | MrpB | MbhF | Sodium translocation | - |  |
| Sodium translocation | MrpC | MbhG | Proton translocation? | Nqo11 | P-module |
| Proton translocation | MrpD | MbhH | Proton  translocation | Nqo13/14 | P-module |
| Sodium translocation | MrpE | MbhA | Sodium translocation | - |  |
| Sodium translocation | MrpF | MbhB | Sodium translocation | - |  |
| Sodium translocation | MrpG | MbhC | Sodium translocation | - |  |

In complex I, N-module represents NADH-oxidising module, Q-module is a connecting module where quinone is reduced and P-module is a proton-pumping membrane module. For MBH, we propose to re-assign the role of MbhDEG subunits to sodium translocation, similarly to Mrp. MA hydrogenase stands for membrane-anchored hydrogenase module.

**Supplementary Table 3**

**Summary of impact of mutations on expression level of Mrp subunits, growth in high salt, antiport activity in everted membrane vesicles, Km values for Na^+^ and assembly of the mutant, as well as of structural environment of the mutated residue.**

^a^ Mutated residue as it was published in the respective reference and the same mutated residue *in italics* according to our own alignment (if different from published reference) using the UNIPROT accession codes B7GL84 for MrpA, B7GL83 for MrpB, B7GL82 for MrpC, B7GL98 for MrpD, B7GL97 for MrpE, B7GL96 for MrpF, B7GIG3 for MrpG from *Anoxybacillus flavithermus* WK1, Q9RGZ5 for MrpA, Q9RGZ4 for MrpB, Q9RGZ3 for MrpC, Q9RGZ2 for MrpD, Q9RGZ1 for MrpE, Q9RGZ0 for MrpF, Q9RGY9 for MrpG from *Bacillus pseudofirmus* OF4, Q9K2S2 for MrpA, O05259 for MrpB, O05260 for MrpC, O05229 for MrpD, Q7WY60 for MrpE, O05228 for MrpF, O05227 for MrpG from *Bacillus subtilis*. For Mrp1 from *Corynebacterium glutamicum* the sequences from EnsemblBacteria CAF18840 for Mrp1A, CAF18839 for Mrp1C, CAF18838 for Mrp1D, CAF18837 for Mrp1E, CAF18836 for Mrp1F and CAF18835 for Mrp1G were used.

^b^ Ratio of the lowest expressed Mrp subunit compared with the positive control which has a value of 1. For references 4 and 6, the protein bands were visually compared with the control band.

^c^ Percent dequenching in everted membrane vesicles as compared with the positive control at pH 8.0 or pH 8.5 and K_m_ values for Na^+^.

^d^ The “+” indicates a growth of KNabc cells in LBK medium containing 400-600 mM NaCl. The percentage values are the means of growth of KNabc cells in LBK medium containing 600 mM NaCl as compared with the control.

^e^ The presence of the Mrp monomer, dimer and MrpA-D subcomplex is indicated as “✓”.

^f^ Expression, antiport activity and growth in NaCl were tested in the antiporter-deficient KNabc *E. coli* strain and/or in *Bacillus subtilis.*

^g^ “Top” means towards the cytoplasm and “bottom” towards the periplasm.

**Abbreviations:**

BS… *Bacillus subtilis*

BP…*Bacillus pseudofirmus* OF4

CG*…Corynebacterium glutamicum*

**Categories:**

1. Effects on monomer/dimer/sub complex
2. Reduced EMV activity
3. Reduced growth in NaCl
4. Normal
5. Km for Na^+^ at least 2.5 fold increase

* The asterisk indicates a contradiction between the antiport activity and the growth in NaCl. Usually the growth in NaCl is a more reliable indicator of antiport activity.

Controls from different references

| Reference | Bacterial species | Control | Expression^b^ | Effect | | | | |
| --- | --- | --- | --- | --- | --- | --- | --- | --- |
|  |  |  |  | Antiport activity^c^ | Growth in NaCl^d^ | Assembly  of the mutant^e^ | | |
| 1 | Bacillus pseudofirmus OF4 | TFCHS7 | 1 | 100%  K_m_ 0.096 | + | D  ✓ | M  ✓ | A-D  ✓ |
| 2 | Bacillus pseudofirmus OF4 | TFCHS7 | 1 | Normal  K_m_ 0.079 | NA | D  ✓ | M  ✓ | A-D  ✓ |
| 3 | Bacillus pseudofirmus OF4 | TFCHS7 | 1 | 100%  K_m_ 0.079 | + | D  ✓ | M  ✓ | A-D  ✓ |
| 4 | Bacillus subtilis | pTY11 | 100% | 100% | 100% | NA | | |
| 5 | Corynebacterium glutamicum | Original mrp1 | NA | NA | + | NA | | |
| 6 | Bacillus subtilis  Bacillus subtilis | pTY11  Δstrains | 100%  KNabc^f^  100%  BS^f^ | 100%  KNabc^f^  100%  BS^f^ | 100%  KNabc^f^  100%  BS^f^ | NA  NA | | |

**MrpA**

| Residue  in *A. flavi-thermus* | Homologous  residue, species, mutation^a^ | Expression^b^ | Effect | | | | | Amino acid location^g^ | Cate-gory | Refe-rence |
| --- | --- | --- | --- | --- | --- | --- | --- | --- | --- | --- |
|  |  |  | Antiport activity^c^ | Growth in NaCl^d^ | Assembly  of the mutant^e^ | | |  |  |  |
| D104 | D50N  *D77N*  BS  D50N  *D77N*  BS | ~100%  ~50%  BS | 12%  56%  BS | >100%  65%  BS | NA  NA  BS | | | Link between βH element and TM8 | 2*  2/3 | 4  6 |
| D104 | D77A  *D78A*  BP  D50A  *D77A*  BS | >0.8  ~90% | 99%  0% | +  71% | D  ✓  NA | M  ✓  NA | A-D  ✓  NA |  | 4  2* | 1  4 |
| D104 | D77E  *D78A*  BP  D50E  *D77E*  BS  D50E  *D77E*  BS | >0.77  ~100%  ~10%  BS | 84%  21%  40%  BS | +  79%  45%  BS | D  ✓  NA  NA | M  ✓  NA  NA | A-D  ✓  NA  NA |  | 4  2*  2/3 | 1  4  6 |
| D157 | D103A  *D130A*  BS | ~20% | 38% | 0% | NA | | | Link between TM5 and βH element | 2/3 | 4 |
| D157 | D103E  *D130E*  BS  D103E  *D130E*  BS | ~50%  ~50%  BS | 0%  64%  BS | 0%  61%  BS | NA  NA | | |  | 2/3  2/3 | 4  6 |
| D157 | D103N  *D130N*  BS  D103N  *D130N*  BS | ~100%  ~50%  BS | 62%  76%  BS | 5%  24%  BS | NA  NA | | |  | 3*  3* | 4  6 |
| Y163 | Y136A  *Y137A*  BP | >1 | 100% | + | D  ✓ | M  ✓ | A-D  ✓ | Near GluTMH5, interface to MrpD | 4 | 3 |
| E167 | E113D  *E140D*  BS  E113D  *E140D*  BS | ~80%  ~50 %  BS | 45%  60%  BS | 7%  1%  BS | NA  NA | | | GluTMH5 | 3  3* | 4  6 |
| E167 | E113Q  *E140Q*  BS  E113Q  *E140Q*  BS | ~80%  ~50%  BS | 0%  20%  BS | 0%  0%  BS | NA  NA | | |  | 2/3  2/3 | 4  6 |
| E167 | E140A  *E141A*  BP  E113A  *E140A*  BS | >0.19  ~70% | 6%  12% | -  0% | D  ✓  NA | M  ✓  NA | A-D  X  NA |  | 1  2/3 | 3  4 |
| K248 | K223A  *K224A*  *BP* | >0.11 | 0% | - | D  X | M  ✓ | A-D  ✓ | LysTMH7 | 1 | 3 |
| Y255 | H230K  *H231A*  BP  H230K  CG | >0.79  NA | 65%  Km 0.25 mM  NA | ±  ± | D  ✓  NA | M  ✓  NA | A-D  ✓  NA | Interaction between TMH7 and HL helix | 5  3 | 3  5 |
| Y255 | H230A  *H231A*  BP | 0.89 | >100% | + | D  ✓ | M  ✓ | A-D  ✓ |  | 4 | 3 |
| W257 | W232A  *H233A*  BP | >0.84 | 86% | + | D  ✓ | M  ✓ | A-D  ✓ | Upper part of TM7 | 4 | 3 |
| Y283 | Y258A  *Y259A*  BP | >1 | 92% | + | D  ✓ | M  ✓ | A-D  ✓ | Lower part of TM8 | 4 | 3 |
| R287 | R262A  *R263A*  BP | >0.87 | 96% | + | D  ✓ | M  ✓ | A-D  ✓ | Salt bridge to D104 | 4 | 1 |
| Q320 | Q295A  *Q296A*  BP | >0.5 | 91% | + | D  ✓ | M  ✓ | A-D  ✓ | Loop between TM9-TM10 | 4 | 1 |
| K324 | K299A  *K300A*  BP | >0.5 | 5% | - | D  ✓ | M  ✓ | A-D  ✓ | Top of TM10,  Possible exit point for H^+^ | 2/3 | 3 |
| K324 | K299H  CG | NA | NA | - | NA | | |  | 3 | 5 |
| G336 | G311A  *G312A*  BP | >0.83 | 97% | + | D  ✓ | M  ✓ | A-D  ✓ | Middle of TM10 | 4 | 1 |
| H369 | H345A  *H346A*  BP | >0.94 | 90%  Km 0.092 mM | + | D  ✓ | M  ✓ | A-D  ✓ | Middle of TM11, part of H^+^ entry cluster | 4 | 3 |
| F381 | F357A  *F358A*  BP | >0.96 | 93% | + | D  ✓ | M  ✓ | A-D  ✓ | Top of TM11 | 4 | 1 |
| G416 | G392A  BP | >0.98 | 100% | + | D  ✓ | M  ✓ | A-D  ✓ | Upper part of TM12 | 4 | 3 |
| G416 | G392R  *G393R*  BP | >0.18 | 3% | - | D  X | M  ✓ | A-D  ✓ |  | 1 | 3 |
| G416 | G378T  CG | NA | NA | + | NA | | |  | 4 | 5 |
| F429 | F405A  *F406A*  BP | >0.9 | 100% | + | D  ✓ | M  ✓ | A-D  ✓ | TM12, next to the break | 4 | 3 |
| H547 | H499K  CG | NA | NA | + | NA | | | Loop between TM14 and TM15 | 4 | 5 |
| F690 | F671A  *F672A*  BP | >0.97 | 79% | + | D  ✓ | M  ✓ | A-D  ✓ | TM18, next to MrpC | 4 | 1 |
| P696 | P677G  *P678G*  BP | >0.91 | 84% | + | D  ✓ | M  X | A-D  ✓ | TM19; periplasmic surface | 1 | 1 |
| T701 | T683A  BP | >0.7 | 87% | + | D  ✓ | M  ✓ | A-D  ✓ | TM19, next to MrpC | 4 | 1 |
| E706 | E687A  *T688A*  BP  E657A  *E684A*  BS | >0.48  ~100% | 0.7%  0% | -  0% | D  ✓  NA | M  ✓  NA | A-D  ✓  NA | TM19, next to MrpC,  5 Å from bound K^+^ | 2/3  2/3 | 1  4 |
| E706 | E657D  *E684D*  *BS*  E657D  *E684D*  *BS* | ~100%  ~70%  BS | 93%  100%  BS | >100%  89%  BS | NA  NA | | |  | 4  4 | 4  6 |
| E706 | E657Q  *E684Q*  *BS*  E657Q  *E684Q*  BS | ~100%  ~70%  BS | 2%  48%  BS | 5%  0%  BS | NA  NA | | |  | 2/3  2/3 | 4  6 |
| H719 | H700A  *H701A*  BP | >0.9 | 19%  Km 0.30 mM | + | D  ✓ | M  ✓ | A-D  ✓ | Loop between TM19 & TM20, coordinates headgroup of lipid bound in Na^+^ cavity | 5 | 3 |
| H719 | H700K  *H701A*  BP | >1 | 56%  Km 0.43 mM | + | D  ✓ | M  ✓ | A-D  ✓ |  | 5 | 3 |
| H719 | H700W  *H701A*  BP | >1 | 63%  Km 0.37 mM | + | D  ✓ | M  ✓ | A-D  ✓ |  | 5 | 3 |
| P721 | P702G  *P703A*  BP | 0.45 | 34%  Km 0.326 mM | + | D  ✓ | M  ✓ | A-D  ✓ | Loop between TM19 & TM20 | 5 | 1 |
| R789 | R773A  *R774A*  BP | >0.18 | 1% | - | NA | | | Loop before TM21; salt bridge to D128*d* | 2/3 | 1 |
| D792 | D743A  *D770A*  BS | ~30% | 0% | 0% | NA | | | Periplasmic end of TM21; exit of Na^+^ pathway | 2/3 | 4 |
| D792 | D743E  *D770E*  BS  D743E  *D770E*  BS | ~100%  ~100%  BS | >100%  >100%  BS | 90%  90%  BS | NA  NA | | |  | 4  4 | 4  6 |
| D792 | D743N  *D770N*  BS  D743N  *D770N*  BS | ~100%  ~90%  BS | 0%  16%  BS | 0%  0%  BS | NA  NA | | |  | 2/3  2/3 | 4  6 |
| E796 | E747D  *E774D*  BS  E747D  *E774D*  BS | ~100%  ~100%  BS | 0%  36%  BS | 10%  2%  BS | NA  NA | | | TM21, facing MrpC;  Part of Na^+^ pathway | 2/3  2/3 | 4  6 |
| E796 | E747Q  *E774Q*  BS  E747Q  *E774Q*  BS | ~100%  ~20%  BS | 0%  4%  BS | 0%  0%  BS | NA  NA | | |  | 2/3  2/3 | 4  6 |
| E796 | E780A  *E781A*  BP  E747A  *E774A*  BS | >0.46  ~100% | 0%  0% | -  0% | D  ✓  NA | M  ✓  NA | A-D  ✓  NA |  | 2/3  2/3 | 1  4 |

**MrpB**

| Residue  in *A. flavi-thermus* | Homologous residue, species, mutation^a^ | Expression^b^ | Effect | | | | | Amino acid location^g^ | Cate-gory | Refe-rence |
| --- | --- | --- | --- | --- | --- | --- | --- | --- | --- | --- |
|  |  |  | Antiport activity^c^ | Growth in NaCl^d^ | Assembly  of the mutant^e^ | | |  |  |  |
| H31 | H34A  BP | >0.9 | >100%  Km 0.092 | + | D  ✓ | M  ✓ | A-D  ✓ | Loop between TM1 & TM2; periplasmic surface | 4 | 3 |
| P34 | P37G  BP | >1 | 83%  Km 0.057 mM | + | D  ✓ | M  X | A-D  ✓ | Loop between TM1 & TM2; periplasmic surface | 1 | 3 |
| F38 | F41A  BP | >1 | 83%  Km 0.14 mM | - | D  ✓ | M  ✓ | A-D  ✓ | TM2 next to MrpA | 3* | 3 |
| D118 | D121A  BS | ~100%  KNabc | 0%  KNabc | 0%  KNabc | NA | | | Periplasmic end of  TM2, interacts with N782*a* | 2/3 | 6 |
|  | D121E  BS  D121E  BS | ~90%  KNabc  ~100%  BS | 18%  KNabc  80%  BS | 84%  KNabc  91%  BS | NA  NA | | |  | 2*  4 | 6  6 |
|  | D121N  BS  D121N  BS | ~70 %  KNabc  ~100%  BS | 10%  KNabc  0%  BS | 0%  KNabc  91%  BS | NA  NA | | |  | 2/3  2* | 6  6 |

**MrpC**

| Residue  in *A. flavi-thermus* | Homologous residue, species, mutation^a^ | Expression^b^ | Effect | | | | | Amino acid location^g^ | Cate-gory | Refe-rence |
| --- | --- | --- | --- | --- | --- | --- | --- | --- | --- | --- |
|  |  |  | Antiport activity^c^ | Growth in NaCl^d^ | Assembly  Of the mutant^e^ | | |  |  |  |
| Q70 | Q70A  BP | 0.85 | 77%  Km 0.068 mM | + | D  ✓ | M  X | A-D  ✓ | Bottom of TM3, interacts with MrpA | 1 | 3 |
| T75 | T75A  BP | >0.86 | 88%  Km 0.11 mM | - | D  ✓ | M  ✓ | A-D  ✓ | TM3; interface with MrpD/A, part of Na^+^ exit pathway | 3* | 3 |
| I77 | I76F  CG | NA | NA | - | NA | | | Middle of TM3, part of cavity where K^+^ ion is bound. | 3 | 5 |
| G82 | G82P  BP | >0.43 | 81%  Km 0.083 mM | + | D  ✓ | M  ✓ | A-D  ✓ | TM3, next to C-terminus of MrpA | 4 | 3 |
| G82 | G82I  BP | >0.7 | 55%  Km 0.12 mM | + | D  ✓ | M  ✓ | A-D  ✓ |  | 2 | 3 |

**MrpD**

| Residue  in *A. flavi-thermus* | Homologous residue, species, mutation^a^ | Expression^b^ | Effect | | | | | Amino acid location^g^ | Cate-gory | Refe-rence |
| --- | --- | --- | --- | --- | --- | --- | --- | --- | --- | --- |
|  |  |  | Antiport activity^c^ | Growth in NaCl^d^ | Assembly  of the mutant^e^ | | |  |  |  |
| D75 | D75E  BP  D75E  BS  D75E  BS | >0.5  ~50 %  KNabc  ~100%  BS | 69%  Km 0.056 mM  9%  KNabc  87%  BS | +  0% KNabc  >100%  BS | D  ✓  NA  NA | M  ✓  NA  NA | A-D  ✓  NA  NA | Link between βH element and TM8 | 2  2/3  4 | 1  6  6 |
| D75 | D75A  BP  D75A  BS | >0.06  ~50%  KNabc | 9%  9%  KNabc | -  0%  KNabc | D  X  NA | M  X  NA | A-D  X  NA |  | 1  2/3 | 1  6 |
| D75 | D75N  BS  D75N  BS | ~80 %  KNabc  ~100%  BS | 15%  KNabc  97%  BS | 0%  KNabc  99%  BS | NA  NA | | |  | 2/3  4 | 6  6 |
| D128 | D128E  BS  D128E  BS | ~100 %  KNabc  ~100 %  BS | 13%  KNabc  81%  BS | >100%  KNabc  >100%  BS | NA  NA | | | Link between TM5 and βH element | 2*  4 | 6  6 |
| D128 | D128A  BS | >50 %  KNabc | 4%  KNabc | 8%  KNabc | NA | | |  | 2/3 | 6 |
| D128 | D128N  BS  D128N  BS | ~100 %  KNabc  ~100%  BS | 15%  KNabc  100%  BS | 89%  KNabc  74%  BS | NA  NA | | |  | 2*  4 | 6  6 |
| F135 | F135A  BP | >0.9 | 97% | + | D  ✓ | M  ✓ | A-D  ✓ | TM5; next to C-term. of MrpA | 4 | 3 |
| F136 | F136A  BP | >0.94 | 75% | + | D  ✓ | M  ✓ | A-D  ✓ | TM5; next to GluTMH5 | 4 | 3 |
| F136 | F136T  BP | >1 | 79% | + | D  ✓ | M  ✓ | A-D  ✓ |  | 4 | 3 |
| F136 | F136E  BP | >1 | 78% | + | D  ✓ | M  ✓ | A-D  ✓ |  | 4 | 3 |
| F136 | F136G  BP | >1 | 37%  Km 0.31 mM | - | D  ✓ | M  ✓ | A-D  ✓ |  | 5 | 3 |
| E137 | E137A  BP  E137A  BS | >0.26  NA  KNabc | 0%  2%  KNabc | -  0%  KNabc | D  X  NA | M  ✓  NA | A-D  X  NA | GluTMH5;  Na^+^ coupling | 2/3  2/3 | 3  6 |
| E137 | E137D  BP  E137D  BS  E137D  BS | >0.84  ~100%  KNabc  100%  BS | 92%  Km 0.22 mM  53%  KNabc  48%  BS | -  >100%  KNabc  80%  BS | D  ✓  NA  NA | M  ✓  NA  NA | A-D  ✓  NA  NA |  | 5  2*  2* | 3  6  6 |
| E137 | E137Q  BP  E137Q  BS  E137Q  BS | NA  ~100 %  KNabc  ~50%  BS | NA  0%  KNabc  0%  BS | -  0%  KNabc  0%  BS | NA  NA  NA | | |  | 3  2/3  2/3 | 3  6  6 |
| K219 | K219A  BP | >0.95 | 0.% | - | D  ✓ | M  ✓ | A-D  ✓ | LysTMH7 | 2/3 | 3 |
| W228 | W228A  BP | >0.96 | 86%  Km 0.063 mM | + | D  ✓ | M  ✓ | A-D  ✓ | Loop of TM7 | 4 | 3 |
| R258 | R258A  BP | >0.01 | 30%  Km 0.064 mM | - | D  X | M  X | A-D  X | TM8;  Salt bridge to D75 | 2/3 | 1 |
| G309 | G309A  BP | >0.5 | 88% | + | D  ✓ | M  ✓ | A-D  ✓ | Middle part of TM10 | 4 | 1 |
| F341 | F341A  BP | >0.5 | 64%  Km 0.463 mM | - | D  ✓ | M  ✓ | A-D  ✓ | Part of the block between cytoplasm and LysTM8 | 5 | 1 |

**MrpE**

| Residue  in *A. flavi-thermus* | Homologous residue, species, mutation^a^ | Expression^b^ | Effect | | | | | Amino acid location^g^ | Cate-gory | Refe-rence |
| --- | --- | --- | --- | --- | --- | --- | --- | --- | --- | --- |
|  |  |  | Antiport activity^c^ | Growth in NaCl^d^ | Assembly  of the mutant^e^ | | |  | A | A |
| Q4 | Q4A  BP | NA | Normal | NA | NA | | | Start of TM1 | 4 | 2 |
| N8 | N8A  BP | NA | Normal | NA | NA | | | TM1, stabilises TM2-AH loop | 4 | 2 |
| K59 | K59A  BP | NA | Normal | NA | NA | | | AH helix; facing the cytoplasm | 4 | 2 |
| K66 | K66A  BP | NA | Normal | NA | NA | | | AH helix; facing the cytoplasm | 4 | 2 |
| E67 | E67A  BP  E67A  BS | NA  ~80%  KNabc | Normal  28%  KNabc | NA  68%  KNabc | NA  NA | | | AH helix; facing the cytoplasm, interacts with ferredoxin-like fold | 4  2* | 2  6 |
| E67 | E67D  BS  E67D  BS | ~100%  KNabc  ~90%  BS | 89%  KNabc  100%  BS | >100%  KNabc  >100%  BS | NA  NA | | |  | 4  4 | 6  6 |
| E67 | E67Q  BS  E67Q  BS | ~100%  KNabc  ~100%  BS | 58%  KNabc  >100%  BS | 97%  KNabc  >100%  BS | NA  NA | | |  | 2  4 | 6  6 |
| T113 | T113A  BP  T113A  BP | >0.07  0.02 | 1%  - | -  NA | D  X  X | M  X  ✓ | A-D  ✓  X | Loop before HH2 helix in ferredoxin-like fold, stabilises the helix | 1  1 | 3  2 |
| T113 | T113Y  BP | >1 | 2% | - | D  ✓ | M  ✓ | A-D  ✓ |  | 2/3 | 3 |
| P114 | P114A  BP | >0.91 | 79%  Km 0.11 mM | + | D  ✓ | M  ✓ | A-D  ✓ | Loop before HH2 helix in ferredoxin-like fold, interacts with AH helix | 4 | 3 |
| P114 | P114G  BP  P114G  BP | >0.95  0.73 | 12%  Km 1.6 mM  -  Km 1.3 | +  NA | D  ✓  ✓ | M  ✓  ✓ | A-D  ✓  ✓ |  | 2*  2 | 3  2 |
| P114 | P61L | NA | NA | + | NA | | |  | 4 | 5 |
| H131 | H131A  BP | NA | Normal | NA | NA | | | On β-strand, part of Na^+^ pathway | 4 | 2 |

**MrpF**

| Residue  in *A. flavi-thermus* | Homologous residue, species, mutation^a^ | Expression^b^ | Effect | | | | | Amino acid location^g^ | Cate-gory | Refe-rence |
| --- | --- | --- | --- | --- | --- | --- | --- | --- | --- | --- |
|  |  |  | Antiport activity^c^ | Growth in NaCl^d^ | Assembly  of the mutant^e^ | | |  |  |  |
| R20 | R35H  CG | NA | NA | + | NA | | | Top of TM1 | 4 | 5 |
| P25 | P28G  BP | >0.97 | 94% | + | D  ✓ | M  ✓ | A-D  ✓ | Loop between TM1 & TM2; cytoplasmic surface | 4 | 3 |
| D29 | D32A  BP | >0.27 | 0% | - | D  X | M  X | A-D  ✓ | Cytoplasmic surface of TM2, salt bridge to R40*g* | 1 | 3 |
| R30 | R33A  BP | >0.81 | 46%  Km 0.082 mM | + | D  ✓ | M  ✓ | A-D  ✓ | Cytoplasmic surface of TM2 | 2 | 3 |
| D35 | D38A  BS | ~5%  KNabc | 1%  KNabc | 0%  KNabc | NA | | | Middle of TM2,  part of Na^+^ pathway | 2/3 | 6 |
| D35 | D38E  BS  D38E  BS | ~40%  KNabc  ~100%  BS | 3%  KNabc  80%  BS | 0%  KNabc  >100%  BS | NA  NA | | |  | 2/3  4 | 6  6 |
| D35 | D38N  BS  D38N  BS | ~40%  KNabc  ~100%  BS | 4%  KNabc  >100%  BS | 0%  KNabc  75%  BS | NA  NA | | |  | 2/3  4 | 6  6 |

**MrpG**

| Residue  in *A. flavi-thermus* | Homologous residue, species, mutation^a^ | Expression^b^ | Effect | | | | | Amino acid location^g^ | Cate-gory | Refe-rence |
| --- | --- | --- | --- | --- | --- | --- | --- | --- | --- | --- |
|  |  |  | Antiport activity^c^ | Growth in NaCl^d^ | Assembly  of the mutant^e^ | | |  |  |  |
| R33 | R28H | NA | NA | + | NA | | | End of TM1 | 4 | 5 |
| P86 | P81G  BP | >1 | 8% | + | D  ✓ | M  ✓ | A-D  ✓ | on a kink in TM3, interacting with MrpE | 2* | 3 |
| P86 | P81A  BP | >0.96 | 8% | + | D  ✓ | M  ✓ | A-D  ✓ |  | 2* | 3 |

**References**

1

Morino, M., Ogoda, S., Krulwich, T. A., & Ito, M. (2017). Differences in the phenotypic effects of mutations in homologous MrpA and MrpD subunits of the multi-subunit Mrp-type Na+/H+ antiporter. *Extremophiles*. https://doi.org/10.1007/s00792-016-0877-z

2

Morino, M., Natsui, S., Swartz, T. H., Krulwich, T. A., & Ito, M. (2008). Single gene deletions of mrpA to mrpG and mrpE point mutations affect activity of the Mrp Na+/H+ antiporter of alkaliphilic Bacillus and formation of hetero-oligomeric Mrp complexes. *Journal of Bacteriology*, *190*(12), 4162–4172. https://doi.org/10.1128/JB.00294-08

3

Morino, M., Natsui, S., Ono, T., Swartz, T. H., Krulwich, T. A., & Ito, M. (2010).

Single site mutations in the hetero-oligomeric Mrp antiporter from alkaliphilic Bacillus pseudofirmus OF4 that affect Na^+^/H^+^ antiport activity, sodium exclusion, individual Mrp protein levels, or Mrp complex formation. *Journal of Biological Chemistry*, *285*(40), 30942–30950. https://doi.org/10.1074/jbc.M110.118661

4

Kosono, S., Kajiyama, Y., Kawasaki, S., Yoshinaka, T., Haga, K., & Kudo, T. (2006). Functional involvement of membrane-embedded and conserved acidic residues in the ShaA subunit of the multigene-encoded Na+/H+ antiporter in Bacillus subtilis. *Biochimica et Biophysica Acta - Biomembranes*. https://doi.org/10.1016/j.bbamem.2006.04.012

5

Xu, N., Zheng, Y., Wang, X., Krulwich, T. A., Ma, Y., & Liu, J. (2018). The lysine 299 residue endows the multisubunit Mrp1 antiporter with dominant roles in Na+ resistance and pH homeostasis in Corynebacterium glutamicum. *Applied and Environmental Microbiology*. https://doi.org/10.1128/AEM.00110-18

6

Kajiyama, Y., Otagiri, M., Sekiguchi, J., Kudo, T., & Kosono, S. (2009). The MrpA, MrpB and MrpD subunits of the Mrp antiporter complex in bacillus subtilis contain membrane-embedded and essential acidic residues. *Microbiology*, *155*(7), 2137–2147. https://doi.org/10.1099/mic.0.025205-0
